# PLLA@PDA-DOX Nanobubbles for Ultrasound Imaging Combined Chemo-Photothermal Therapy

**DOI:** 10.3390/biom16060834

**Published:** 2026-06-04

**Authors:** Jie Zhang, Xinyi Li, Huiming Zhang, Mingzhong Wu, Baoqing Gao, Da Zhang, Hongyun Cui

**Affiliations:** Jiamusi University, Jiamusi 154007, China; zhangjie@jmsu.edu.cn (J.Z.); lixy1280@163.com (X.L.); zhmjms@jmsu.edu.cn (H.Z.); wumingzhong1968@163.com (M.W.); 13837424110@163.com (B.G.); zhangda@jmsu.edu.cn (D.Z.); cuihongyun@jmsu.edu.cn (H.C.)

**Keywords:** ultrasonic contrast agent, photothermal therapy, polydopamine, doxorubicin

## Abstract

The photothermal conversion capability of polydopamine (PDA) was exploited to load the anticancer drug doxorubicin (DOX) onto its surface via π-π stacking and hydrogen-bond interactions, yielding a PDA-DOX complex. In this study, biocompatible poly-L-lactic acid (PLLA) was employed as a shell material to fabricate multifunctional PLLA composite PDA-DOX (PLLA@PDA-DOX) nanobubbles with integrated functions of ultrasound imaging, photothermal therapy, and chemotherapy. The fabricated nanobubbles exhibited a uniform mean diameter of 489.30 ± 6.96 nm with a Polydispersity index (PDI) of 0.226 ± 0.01 and a DOX loading efficiency of 3.27%. Acute toxicity evaluation in mice revealed that the maximum tolerated dose of PLLA@PDA-DOX nanobubbles was markedly higher than the clinical equivalent dose, showing no detectable toxicity or allergic reactions. Under near-infrared (NIR) laser irradiation, the inhibition rate of HCCLM3 cells increased from 50.1% to 64.45%, indicating enhanced therapeutic efficacy through the combined effects of photothermal therapy and chemotherapy. Moreover, compared with the free DOX group, the survival rate of LX-2 cells in the composite nanobubble group significantly increased from 18.9 ± 1.56% to 68.8 ± 3.08%, suggesting that the PLLA@PDA-DOX nanobubbles effectively reduced the direct cytotoxicity of DOX by preventing its immediate contact with cells. Collectively, the results confirm that PLLA@PDA-DOX nanobubbles possess excellent biocompatibility, robust ultrasound imaging performance, and enhanced antitumor efficacy under NIR irradiation. This multifunctional nanosystem demonstrates promising potential as an integrated platform for simultaneous cancer diagnosis and therapy.

## 1. Introduction

Primary hepatocellular carcinoma (HCC) is one of the most common malignant tumors worldwide, ranking sixth in incidence and third in cancer-related mortality, which poses a serious threat to global public health [1,2]. Due to the lack of obvious clinical manifestations at the early stage, HCC is often diagnosed at an advanced stage with rapid progression and metastasis, leading to poor prognosis and limited therapeutic efficacy [3,4]. Therefore, the development of effective diagnostic and therapeutic strategies for HCC remains an important challenge in current biomedical research.

Ultrasound imaging has been widely used in clinical diagnosis because of its non-invasive nature, real-time imaging capability, and relatively low cost [5]. However, conventional ultrasound imaging still suffers from limited sensitivity and insufficient image contrast in deep tissues [6]. Ultrasound contrast agents (UCAs) can significantly improve ultrasonic imaging quality by enhancing acoustic backscatter under ultrasonic irradiation [7,8]. Traditional nanobubble-based UCAs are limited by their large particle size, short circulation time, and poor vascular permeability, which restrict their applications in tumor diagnosis and therapy [9]. In contrast, nanoscale ultrasound contrast agents exhibit superior tissue penetration, prolonged circulation behavior, and enhanced permeability and retention (EPR) effects, demonstrating considerable potential in ultrasound molecular imaging, targeted drug delivery, and theranostic applications [10,11,12,13].

PLLA is a biodegradable and biocompatible synthetic polymer that undergoes hydrolytic degradation in vivo, initially producing lactic acid and oligomeric intermediates, which are subsequently metabolized into carbon dioxide and water without generating significant toxic byproducts [14]. Due to its excellent biocompatibility and controllable degradation properties, PLLA has been extensively utilized in biomedical applications, including tissue engineering scaffolds, drug delivery systems, electrospun nanofibers, and injectable microspheres [15,16]. PDA formed through the oxidative self-polymerization of dopamine under alkaline conditions, possesses abundant catechol and amine functional groups that facilitate secondary surface modification and drug immobilization through π–π stacking and hydrogen bonding interactions. Moreover, PDA exhibits remarkable photothermal conversion capability under NIR irradiation and excellent biocompatibility, making it a promising material for photothermal therapy and multifunctional drug delivery systems [17,18,19,20].

Although PDA-based nanoplatforms have demonstrated promising performance in drug delivery and photothermal therapy, several limitations still exist, including poor structural integration between biodegradable carriers and photothermal agents, insufficient control over drug release behavior, and limited therapeutic integration in ultrasound-guided cancer therapy [21,22,23]. In addition, few studies have combined biodegradable PLLA structures with PDA-mediated photothermal systems for simultaneous ultrasound imaging enhancement and combined chemo-photothermal therapy [24,25,26]. Compared with single PLLA-based nanobubbles, the developed PLLA@PDA-DOX nanobubbles primarily serve as ultrasound contrast agents while simultaneously functioning as a drug delivery system for combined PDA-mediated photothermal therapy and DOX chemotherapy within a single theranostic platform. The PLLA shell not only facilitates PDA surface modification and DOX loading through π–π stacking and hydrogen bonding interactions, but also contributes to favorable ultrasound responsiveness and imaging performance. In addition, the incorporation of PDA as an intermediate functional layer endows the nanobubbles with excellent photothermal conversion capability and improved biocompatibility while reducing the cytotoxicity of free DOX toward normal cells. Therefore, this multifunctional nanobubble platform provides a promising strategy for ultrasound imaging-guided combined chemo-photothermal therapy.

In this study, PLLA nanobubbles were successfully fabricated and modified with PDA to construct a multifunctional theranostic platform integrating ultrasound imaging, photothermal therapy, and chemotherapy. DOX was subsequently loaded onto the PLLA@PDA platform to obtain PLLA@PDA-DOX nanobubbles with combined drug delivery and photothermal therapeutic functions. The physicochemical properties, drug loading performance, photothermal behavior, ultrasound imaging performance, and antitumor activity of the developed system were systematically investigated through both in vitro and in vivo experiments. This study provides a promising strategy for ultrasound-responsive chemo-photothermal combined therapy of hepatocellular carcinoma.

## 2. Experiments

### 2.1. Preparation and Characterization Analysis of PLLA@PDA-DOX Nanobubbles

#### 2.1.1. Preparation of PDA and PDA-DOX Complex

Preparation of PDA: A total of 120 mg of DA was dissolved in Tris–HCl buffer (pH 8.5) and subjected to a self-polymerization reaction at room temperature for 12 h under aerobic conditions in the absence of light. The resulting suspension was repeatedly washed with distilled water and then centrifuged, and the precipitate was collected to obtain the PDA suspension. The PDA suspension was subsequently subjected to a freeze-drying process, with the objective of obtaining PDA powder.

Preparation of PDA-DOX complex: A total of 20 mg of PDA and 30 mg of DOX was accurately weighed and dissolved in 20 mL of anhydrous ethanol. The mixture was transferred to a flask and stirred at 45 °C for 6 h. The PDA-DOX complex was obtained after filtration and drying.

#### 2.1.2. Preparation of PLLA@PDA-DOX Nanobubbles

A total of 0.6 g of PLLA was dissolved in 10 mL of dichloromethane, after which 0.6 mL of PDA-DOX dispersion (10 mg·mL^−1^) was added to 0.5 mL of the internal aqueous phase. The primary emulsion was formed by ultrasonication at 600 W for 5 min under a nitrogen atmosphere. The primary emulsion was then mixed with 30 mL of 2% (*w*/*v*) PVA solution, and the secondary emulsion was produced by ultrasonication shock for 5 min. 40 mL of 6% (*v*/*v*) aqueous isopropyl alcohol solution was added to the secondary emulsion, and the dichloromethane was completely evaporated by magnetic stirring at room temperature. After the precipitate was repeatedly washed with distilled water, centrifuged, and lyophilized, PLLA@PDA-DOX nanobubble powders were obtained.

#### 2.1.3. Determination of Drug Content in PLLA@PDA-DOX Nanobubbles

Ethanolic standard solutions of DOX with concentrations of 2.5, 5, 10, 20, 30, 40 and 50 μg·mL^−1^ were prepared, and anhydrous ethanol was used as the blank control. The absorbance of each standard solution was measured at 480 nm using a UV–visible spectrophotometer (L5S, Shanghai Yidian Analytical Instrument Co., Ltd., Shanghai, China). A calibration curve was then constructed by linear regression, with DOX concentration (μg mL^−1^) on the *x*-axis and absorbance (A) on the *y*-axis, and the linear regression equation for DOX was obtained.

A total of 20 mg of lyophilized PLLA@PDA-DOX nanobubbles was dispersed in 20 mL of anhydrous ethanol and sonicated for 2 h to release the loaded drug. The filtrate obtained after filtration was diluted to 20 mL with anhydrous ethanol, and its absorbance was measured at 480 nm using a UV–visible spectrophotometer. The drug loading efficiency (LE) of DOX was calculated according to Formula (1) based on the DOX calibration curve.

(1) $$\mathrm{Dox}\;\mathrm{loading}\;\mathrm{rate}=\frac{\mathrm{DOX}\;\mathrm{concentration}}{\mathrm{concentration}\;\mathrm{of}\;\mathrm{the}\;\mathrm{composite}\;\mathrm{nanobubbles}}\times 100\%$$

#### 2.1.4. Stability of PLLA@PDA-DOX Nanobubbles

The long-term storage stability of PLLA@PDA-DOX nanobubbles was evaluated using the average particle size as the reference index. The suspension of PLLA@PDA-DOX nanobubbles was stored at −4 °C and sampled on days 0, 7, 10, 14, 20, 30 and 35. The average particle size of the composite nanobubbles was determined using a nanoparticle size analyzer (Zetasizer Nano ZSE, Malvern Instruments Ltd., Malvern, UK), and the average particle size and PDI were plotted as functions of storage time.

#### 2.1.5. Photothermal Conversion of PLLA@PDA-DOX Nanobubbles

Following a cycle of 12 min irradiation and 10 min pause, an 808 nm NIR laser with an output power density of 1 W cm^−2^ was applied to irradiate the suspension of PLLA@PDA-DOX nanobubbles after ultrasonic disruption. The procedure was repeated three times. The temperature variation in the aqueous suspension was monitored and recorded every minute.

### 2.2. Evaluation of the Inhibitory Effect of PLLA@PDA-DOX Nanobubbles on HCCLM3 Cells

The inhibitory effect of PLLA@PDA–DOX nanobubbles on HCCLM3 cells was evaluated using the Cell Counting Kit-8 (CCK-8) assay (CCK-8, Aipubi Biotechnology Co., Ltd., Suzhou, China). WST-8, the main component of the CCK-8 assay, is reduced by mitochondrial dehydrogenases in viable cells to form a water-soluble orange-yellow formazan dye. The absorbance at 450 nm, measured using a microplate reader, indirectly reflects the number of viable cells [27].

#### 2.2.1. Inhibition of the Composite Nanobubbles with Different Concentration on HCCLM3 Cells

Suspensions of PLLA@PDA-DOX nanobubbles at concentrations of 1, 5, 10, 15 and 20 mg mL^−1^ were prepared in MEM complete medium. Five experimental groups and one control group (cells only) were seeded into 48-well plates at a density of 2 × 10^4^ HCCLM3 cells per well. After 24 h of incubation, the medium was removed, and the cells were washed with PBS. Then, 200 μL of composite nanobubble suspensions at different concentrations were added to each well and cultured for another 24 h. Cell viability was assessed using the CCK-8 assay after co-culture. Afterward, 200 μL of CCK-8 working solution was added to each well, and the cells were incubated for 1 h. The reaction solution from each well was transferred to a 96-well microplate, and the absorbance was measured at 450 nm using a microplate reader. The cell growth inhibition rate was calculated according to Formula (2).

(2) $$\mathrm{Cell}\;\mathrm{inhibition}\;\mathrm{rate}\;=\frac{\mathrm{blank}\;\mathrm{group}-\mathrm{experimental}\;\mathrm{group}}{\mathrm{blank}\;\mathrm{group}\text{-}\mathrm{CCK}8}\times 100\%$$

#### 2.2.2. Inhibitory Effects of Different Composite Nanobubbles on HCCLM3 Cells

CCK-8 method: Suspensions of PLLA nanobubbles, PLLA@PDA composite nanobubbles, and PLLA@PDA-DOX nanobubbles were prepared at concentrations of 16.96 mg mmL^−1^, while the free DOX solution was prepared at 0.48 mg mL^−1^ in MEM complete medium. Two sets of 48-well plates were prepared, each containing four experimental groups: PLLA nanobubbles, PLLA@PDA composite nanobubbles, PLLA@PDA-DOX nanobubbles, and free DOX. Four replicate wells were assigned to each group. HCCLM3 cells (2 × 10^4^ cells per well) were seeded into the plates and incubated for 24 h. In one set of plates, cell viability was evaluated using the CCK-8 assay after 24 h of co-culture. In the parallel set of plates, the four groups were irradiated with an 808 nm near-infrared laser at an output power density of 1 W cm^−2^ following a cycle of 10 min irradiation and 10 min pause, repeated three times. Cell viability was then assessed using the CCK-8 assay after 24 h of co-culture. The temperature variation in the PLLA@PDA-DOX nanobubble group was monitored and recorded every minute.

EdU method: HCCLM3 cells in the logarithmic growth phase were seeded into 48-well plates at a density of 2 × 10^4^ cells per well in 200 μL of MEM complete medium and incubated for 24 h. After removing the medium, 200 μL of PLLA nanobubble suspension, PLLA @PDA nanobubble suspension, PLLA@PDA-DOX nanobubble suspension, and free DOX solution (prepared as described in Section 2.1) was added to each well, followed by incubation for 24 h. Afterward, 1 mL of 10 μM EdU working solution was added to each well and incubated for 2 h. The cells were then fixed with 4% paraformaldehyde, washed with 3% BSA–PBS solution, and permeabilized with 1 mL of 0.3% Triton X-100 buffer to increase membrane permeability. Proliferating cells were stained with the click reaction solution, and nuclei were counterstained with the Hoechst dye. The effects of each nanobubble formulation on HCCLM3 cell proliferation were observed using laser confocal microscopy, and the proliferation rate was calculated according to Formula (3).

(3) Cell proliferation rate = Number of proliferating cells/total number of cells

### 2.3. Safety Experiment of PLLA@PDA-DOX Nanobubbles

#### 2.3.1. Cytotoxicity Test

The effects of different nanobubble formulations on the viability of LX-2 cells were evaluated using the CCK-8 assay. LX-2 cells were used as the experimental model. Suspensions of PLLA nanobubbles, PLLA@PDA nanobubbles, and PLLA@PDA-DOX nanobubbles (16.96 mg mL^−1^) as well as a free DOX solution (0.48 mg mL^−1^) were prepared using LX-2 cell culture medium. LX-2 cells were seeded into 48-well plates (2 × 10^4^ cells per well) and incubated for 24 h. The various experimental formulations were then added and co-cultured with the cells for an additional 24 h. Cell viability was calculated according to Formula (4), and cell morphology in each group was observed and photographed using an inverted microscope.

(4) $$\mathrm{Cell}\;\mathrm{survival}\;\mathrm{rate}\;=\frac{\mathrm{Experimental}\;\mathrm{group}\;-\mathrm{CCK}8\;\mathrm{group}\;}{\mathrm{Blank}\;\mathrm{group}\text{-}\mathrm{CCK}8\;\mathrm{group}}\times 100\%$$

The effects of different nanobubble formulations on the proliferation rate of LX-2 cells were evaluated using the EdU assay. After staining with the EdU Cell Proliferation Kit, the effects of PLLA nanobubble suspension, PLLA@PDA composite nanobubble suspension, PLLA@PDA-DOX nanobubble suspension, and free DOX on LX-2 cell proliferation were observed using laser confocal microscopy (FV1000, Olympus Optical Co., Ltd., Tokyo, Japan). The proliferation rate of LX-2 cells was calculated according to Formula (3).

#### 2.3.2. Hemolysis Test

Fresh rabbit blood (5 mL) was collected and anticoagulated with heparin, followed by low-speed centrifugation to obtain erythrocytes. The red blood cells were washed three times with an equal volume of 0.9% NaCl solution. Subsequently, 1 mL of the washed erythrocytes was diluted with 0.9% NaCl solution to a final volume of 50 mL.

Five groups were prepared: PLLA nanobubbles, PLLA@PDA nanobubbles, PLLA@PDA-DOX nanobubbles, normal saline (negative control), and distilled water (positive control). The samples were mixed according to the proportions listed in Table S1 and incubated at 37 °C for 3 h. After incubation, the samples were centrifuged at 3000 rpm for 5 min, and 200 μL of the supernatant from each group was transferred to a 96-well plate.

The optical density (OD) was measured at 545 nm using a microplate reader. The hemolysis rate was calculated according to Formula (5), where ODE, ODNC, and ODPC represent the optical densities of the experimental group, negative control, and positive control, respectively. A hemolysis rate below 5% was considered indicative of non-hemolytic behavior.

(5) Hemolysis rate (%) = (ODE − ODNC)/(ODPC − ODNC) × 100%

#### 2.3.3. Acute Toxicity Test

Nine specific-pathogen-free (SPF) Kunming (KM) mice were randomly divided into three groups with three mice per group. Three dose levels were established: low (1000 mg kg^−1^), medium (2000 mg kg^−1^), and high (4000 mg kg^−1^). PLLA@PDA-DOX nanobubbles (0.1 mL per 10 g body weight) were administered intraperitoneally, and clinical signs and mortality were recorded over 72 h. The experiment was repeated with adjusted dose ratios until both the minimum lethal dose (Dn) and the maximum non-lethal dose (Dm) were determined.

Five dose groups were established within the range of Dn and Dm—2400, 2700, 3000, 3300 and 3600 mg kg^−1^—with six mice randomly assigned to each group. Mice were randomly assigned to five groups (six per group, with equal numbers of males and females) using a two-step randomization method. Prior to the experiment, mice were fasted for 12 h with free access to water. Body weights did not differ by more than ±20% from the group mean.

The high-dose administration used in this study was intended to evaluate the acute toxicity profile and biosafety margin of the developed nanobubbles rather than to simulate clinically relevant therapeutic dosing conditions. Given that PLLA@PDA-DOX nanobubbles are intended for clinical diagnostic use with a relatively low application dose, the approximate Dn value was used as the maximum dose for the safety limit test in mice. Ten KM mice (five males and five females) were randomly selected and injected intraperitoneally with PLLA@PDA-DOX nanobubble suspension at the maximum dose (same volume per body weight). Behavioral changes, clinical signs, and mortality were monitored and recorded for 14 days post-administration. The maximum tolerated multiple was calculated according to Formula (6). For reference, the typical clinical dosage of ultrasound contrast agents is approximately 0.01 g kg^−1^ [28].

(6) $$Maximum\;tolerance\;multiple\;of\;mice=\frac{Tolerance\;dose\;of\;mice}{The\;average\;weight\;of\;the\;mice\;(20\;g)}\times \frac{The\;average\;weight\;of\;an\;adult\;(50\;Kg)}{A\;daily\;dose\;for\;an\;adult}$$

#### 2.3.4. Biocompatibility Test

Ten KM mice were randomly divided into two groups. The control group received 0.2 mL of normal saline, whereas the treatment group was administered 0.2 mL of PLLA@PDA-DOX nanobubble suspension (10 mg·mL^−1^). Local reactions at the intramuscular injection site were visually examined for signs of congestion, edema, or necrosis. At 2, 4, 10, 21 and 30 days post-administration, the mice were euthanized by cervical dislocation. Muscle tissue from the injection site was excised and fixed in 4% paraformaldehyde solution. Tissue samples from each group were embedded in paraffin, sectioned, and stained with hematoxylin and eosin (H&E). The stained sections were examined under a light microscope at 200× magnification.

### 2.4. Ultrasonic Imaging Effect of PLLA Composited PDA-DOX Nanobubbles

In vitro ultrasonic detection: A transparent container was used as a water tank, with a sponge layer placed at the bottom to stabilize the setup. A silicone tube was positioned inside the tank, with both ends extending above the liquid surface. Samples from each group were injected into one end of the silicone tube and repeatedly aspirated to ensure uniform dispersion. Using 0.9% NaCl as the control, the in vitro ultrasonic imaging properties of PLLA nanobubbles, PLLA@PDA composite nanobubbles, and PLLA@PDA-DOX nanobubbles (1 mg mL^−1^) were evaluated using a color Doppler ultrasound imaging system (S40, SIUI, Shantou, China) operating in harmonic imaging mode at 4.5 MHz.

In vivo ultrasonic detection: A healthy 2 kg New Zealand white rabbit was used as the experimental subject. Using 0.9% NaCl as the control, suspensions of PLLA nanobubbles, PLLA@PDA composite nanobubbles, and PLLA@PDA-DOX nanobubbles (1 mg mL^−1^) were injected into the marginal ear vein, and the ultrasound imaging performance of the liver was observed using the S40 color Doppler ultrasound imaging system (SIUI, China) operating in harmonic imaging mode at 4.5 MHz.

### 2.5. Statistical Analysis

All experiments were performed at least in triplicate, and the results are presented as mean ± standard deviation (SD). Statistical analysis was performed using Origin 2021 software (OriginLab Corporation, Northampton, MA, USA). Differences between groups were analyzed using one-way analysis of variance (ANOVA). A value of *p* < 0.05 was considered statistically significant, while * *p* < 0.01 was considered highly statistically significant.

## 3. Results and Discussions

### 3.1. Preparation Results of PLLA@PDA-DOX Nanobubbles

**Figure 1:**
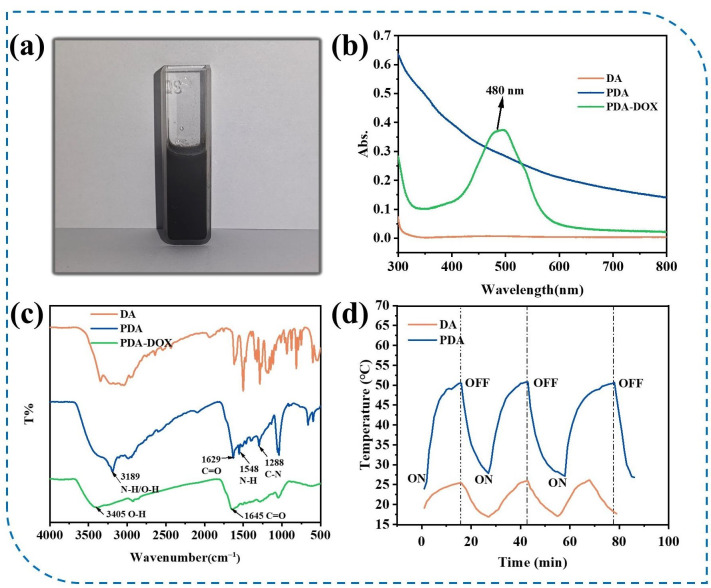
(**a**) Appearance of the PDA aqueous solution; (**b**) UV-vis absorption spectra of DA, PDA, and the PDA-DOX complex; (**c**) FTIR spectra of DA, PDA, and the PDA-DOX complex; (**d**) temperature variation in DA and PDA aqueous solutions under NIR laser irradiation over time.

The PDA aqueous solution was synthesized through oxidative self-polymerization of the DA monomer. The resulting PDA solution appeared light brown, as shown in Figure 1a. Figure 1b presents the ultraviolet–visible (UV-Vis) absorption spectra of DA, PDA, and the PDA-DOX complex. As shown in Figure 1b, DA exhibited almost no absorption in the 300–800 nm region. Compared with DA, PDA displayed strong absorption in the ultraviolet, visible, and near-infrared regions, confirming the successful polymerization of PDA. The PDA-DOX complex also exhibited strong light absorption capacity, with a characteristic DOX peak at 480 nm, indicating successful DOX loading onto PDA.

Figure 1c presents the FT-IR spectra of DA, PDA and the PDA-DOX complex. As shown in Figure 1c, the characteristic peaks corresponding to γ C-N stretching (1288 cm^−1^), β N-H bending (1548 cm^−1^), and catechol O–H stretching (3189 cm^−1^) confirmed that DA was successfully polymerized into PDA. The O–H stretching peak at 3405 cm^−1^ and the C=O stretching peak at 1645 cm^−1^ are characteristic of DOX, confirming the successful loading of DOX onto PDA.

Figure 1d shows the temperature variation curves of DA and PDA aqueous solutions under laser irradiation. As shown in Figure 1d, after 20 min of irradiation in the first cycle, the temperature of the DA aqueous solution increased from 19.3 °C to 25.4 °C. In contrast, the temperature of the PDA aqueous solution increased from 23.9 °C to 50.7 °C, demonstrating its excellent photothermal conversion performance. As observed in Figure 1b,c, PDA exhibited broad absorption across the ultraviolet, visible, and near-infrared regions, which accounts for its strong photothermal conversion ability. Furthermore, after three irradiation cycles, the heating efficiency of the PDA aqueous solution showed minimal change, indicating good photothermal stability and reproducible photothermal performance under repeated laser exposure.

**Figure 2:**
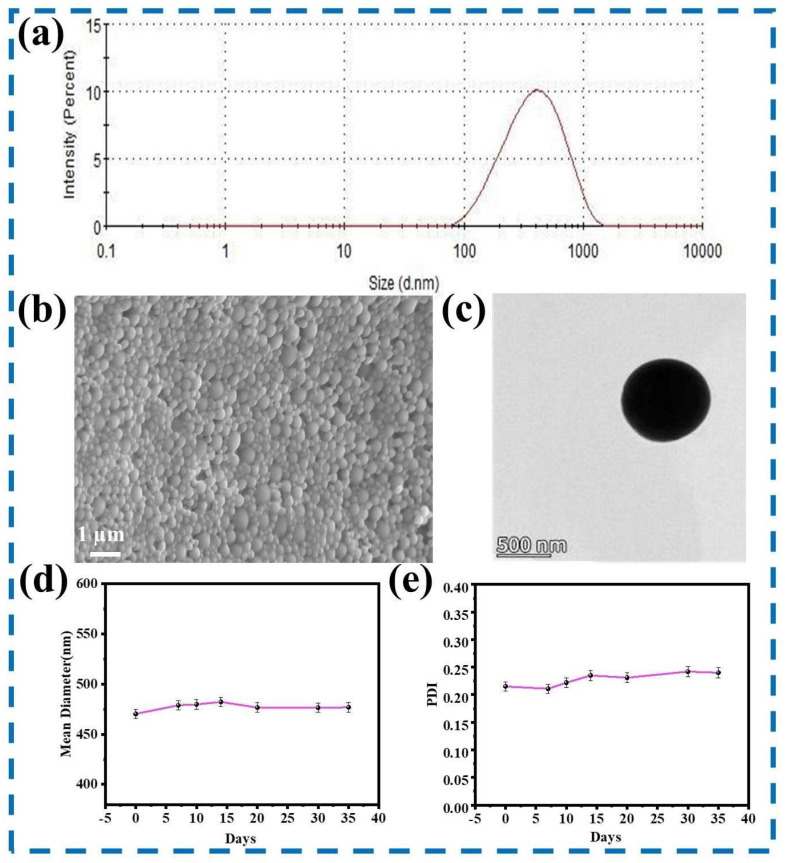
(**a**) Particle size distribution; (**b**) SEM image; (**c**) TEM image; (**d**) average particle size variation; and (**e**) PDI variation in PLLA@PDA-DOX nanobubbles. Data in (**d**,**e**) are presented as mean ± SD (n = 3).

As shown in Figure 2a, the PLLA@PDA-DOX nanobubbles exhibited a uniform and unimodal particle size distribution, with an average diameter of 489.30 ± 6.96 nm and a PDI of 0.226 ± 0.01. Such uniform particle size distribution is favorable for the accumulation of composite nanobubbles at tumor sites. The morphological characteristics of the nanobubbles were further examined by electron microscopy. As presented in Figure 2b,c, the PLLA@PDA-DOX nanobubbles displayed a spherical and regular morphology with smooth, non-adhesive surfaces and relatively uniform size distribution.

The storage stability of the nanobubbles was evaluated by monitoring the changes in particle size and PDI over time. As shown in Figure 2d,e, no significant variation in the average particle size or PDI was observed during 35 days of storage at 4 °C (*p* > 0.05), indicating good storage stability of the PLLA@PDA-DOX nanobubbles.

**Figure 3:**
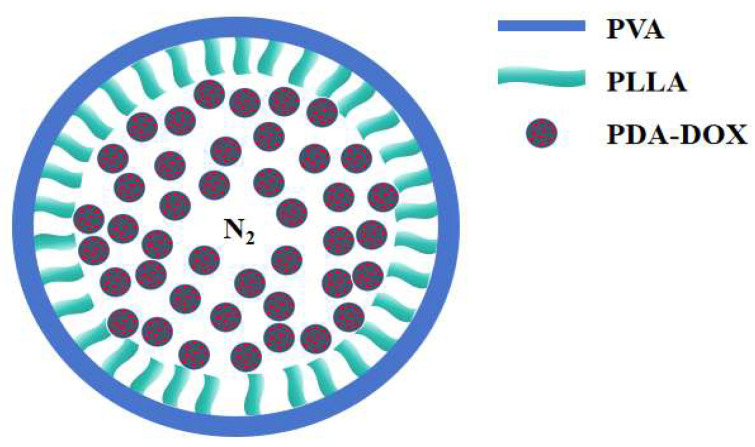
Structural simulation diagram of PLLA@PDA-DOX nanobubbles.

A schematic diagram of the PLLA@PDA–DOX nanobubble structure is shown in Figure 3. During preparation, DOX was loaded onto the PDA surface via π-π stacking or hydrogen bonding interactions. The resulting PDA–DOX aqueous solution served as the internal water phase, while PLLA, dissolved in dichloromethane, acted as the oil phase. During the first ultrasonic emulsification, PLLA was dispersed and fragmented, subsequently encapsulating the PDA–DOX complex to form the primary emulsion. With continuous N_2_ introduction during ultrasonication, PLLA molecules were adsorbed at the gas–liquid interface. Due to the non-polar nature of nitrogen gas, the relatively hydrophobic PLLA domains preferentially oriented toward the gas core, while the relatively hydrophilic groups faced the surrounding solvent phase, thereby contributing to the formation and stabilization of the nanobubbles. The primary emulsion was then transferred into a PVA solution for a second ultrasonic emulsification. PVA, serving as the stabilizer of the external aqueous phase, accumulated and coated the surface of PLLA nanobubbles under ultrasonication, thereby forming the secondary emulsion. The secondary emulsion was transferred into an isopropanol solution, where dichloromethane was volatilized under stirring. The nanobubbles were then freeze-dried, removing residual water. Driven by surface tension, the black PDA–DOX complex became embedded within the membrane and inner layers of the nanobubbles, as illustrated in Figure 2c.

The drug loading content of DOX in PLLA@PDA-DOX nanobubbles was determined based on a standard calibration curve (Figure S1). The calibration curve exhibited a good linear relationship between DOX concentration and absorbance. According to the absorbance of the filtrate and Formula (1), the average drug loading rate was calculated to be 3.27% (Table S2). Although the loading percentage was relatively low, PLLA@PDA-DOX nanobubbles are composed of high-molecular-weight polymeric materials, allowing a considerable amount of DOX to be incorporated into the overall system. More importantly, the present formulation was optimized to achieve a balance between therapeutic efficacy, photothermal performance, structural stability, and biosafety rather than maximizing drug loading alone. The relatively low DOX loading content may also help reduce systemic toxicity while still maintaining effective antitumor activity under combined chemo-photothermal treatment.

**Figure 4:**
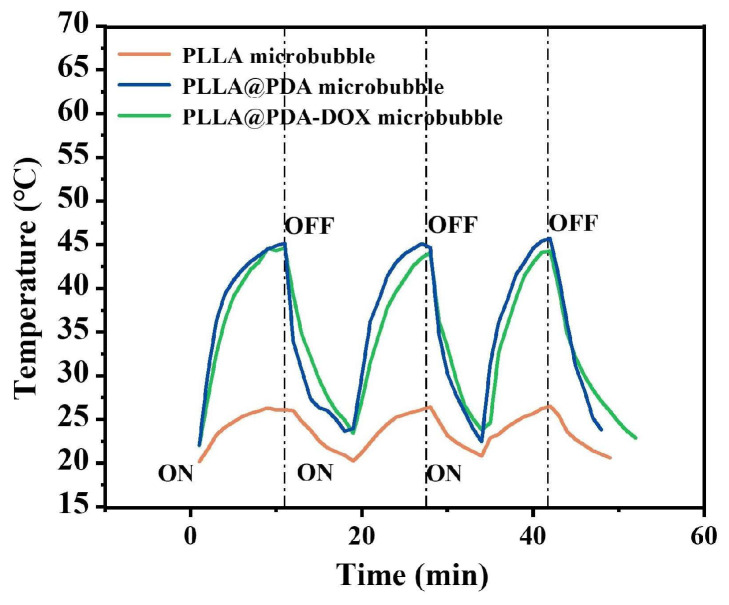
Temperature variation curves of PLLA nanobubbles, PLLA@PDA nanobubbles, and PLLA@PDA-DOX nanobubbles under NIR laser irradiation for three irradiation cycles.

Figure 4 presents the temperature variation curves of PLLA nanobubble suspension, PLLA@PDA composite nanobubble suspension, and PLLA@PDA-DOX nanobubble suspension under NIR laser irradiation. As shown in Figure 4, after 10 min of irradiation in the first cycle, the temperature of the PLLA nanobubble suspension increased slightly from 20.1 °C to 26.4 °C, indicating its limited photothermal conversion capability. In contrast, the temperature of the PLLA@PDA composite nanobubble suspension increased from 22.0 °C to 45.7 °C, while that of the PLLA@PDA-DOX nanobubble suspension increased from 22.8 °C to 44.6 °C, demonstrating that the incorporation of PDA significantly enhances the photothermal conversion efficiency of the nanobubbles.

Notably, the temperature of the PLLA@PDA-DOX nanobubble suspension exceeded 43 °C under NIR irradiation, which is sufficient to induce effective tumor cell ablation. Moreover, after three irradiation cycles, the heating efficiency of both PLLA@PDA and PLLA@PDA-DOX nanobubble suspensions remained nearly unchanged, indicating that DOX loading does not compromise their photothermal performance and that the system possesses good photothermal stability. Previous studies have demonstrated that temperatures above 42–43 °C are generally sufficient to induce irreversible tumor cell damage through protein denaturation, membrane disruption, and apoptosis under photothermal therapy conditions [29,30]. In the present study, the temperature of PLLA@PDA-DOX nanobubbles increased to 44.6 °C under NIR irradiation, indicating that the developed system reached the effective thermal threshold for photothermal tumor ablation. In addition, the stable temperature elevation observed over repeated irradiation cycles further suggests favorable photothermal stability of the PDA-modified nanobubbles.

The enhanced photothermal effect can be attributed to the strong NIR absorption capability of PDA, which enables efficient conversion of light energy into heat. This localized hyperthermia can effectively damage tumor cells while minimizing harm to surrounding normal tissues [31]. Therefore, the PLLA@PDA-DOX nanobubbles exhibit promising potential for combined chemo-photothermal cancer therapy.

### 3.2. Adjuvant Treatment Effect of PLLA@PDA-DOX Nanobubbles

**Figure 5:**
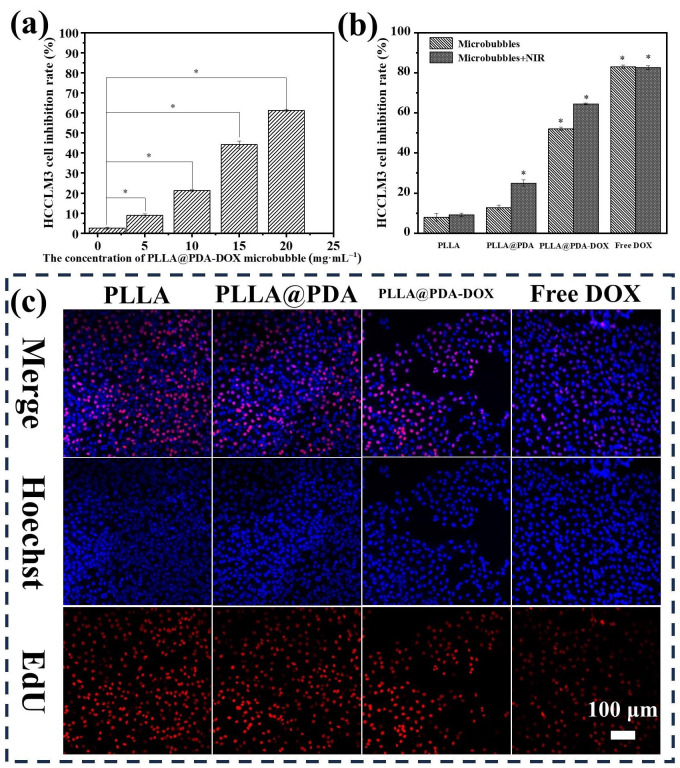
(**a**) Effect of different concentrations of PLLA@PDA-DOX nanobubbles on the proliferation of HCCLM3 cells (* *p* < 0.01 vs. 1 mg·mL^−1^ group). (**b**) Inhibitory effects of different formulations on HCCLM3 cells with or without NIR irradiation (* *p* < 0.01 vs. PLLA nanobubble group). Data are presented as mean ± SD (n = 3) (**c**) EdU staining images of HCCLM3 cells after treatment with different composite nanobubbles: PLLA, PLLA@PDA, PLLA@PDA-DOX, and free DOX.

The inhibition effect of PLLA@PDA-DOX nanobubbles on HCCLM3 cells was first evaluated at different concentrations, and the results are presented in Figure 5a. The inhibition rate was calculated according to Formula (2). As shown in Figure 5a, PLLA@PDA-DOX nanobubbles effectively suppressed the proliferation of HCCLM3 cells in a concentration-dependent manner. Specifically, the inhibition rates at concentrations of 5, 10, 15 and 20 mg mL^−1^ were significantly higher than that at 1 mg mL^−1^, demonstrating a clear dose-dependent inhibitory effect. The IC_50_ value of PLLA@PDA-DOX nanobubbles against HCCLM3 cells was calculated to be 16.96 mg mL^−1^ using IBM SPSS Statistics 27.

The cytotoxic effects of different formulations on HCCLM3 cells were further compared, as shown in Figure 5b. After 24 h of incubation, the inhibition rates of the free DOX group and the PLLA@PDA-DOX nanobubble group were 82.9% and 52.1%, respectively, both significantly higher than that of the PLLA nanobubble group (* *p* < 0.01). In contrast, PLLA@PDA composite nanobubbles exhibited only weak inhibitory activity, with no significant difference compared to the PLLA nanobubble group. Under NIR laser irradiation, the inhibitory effects of PLLA@PDA and PLLA@PDA-DOX nanobubbles were further enhanced. Specifically, the inhibition rates increased from 12.79% to 25.0% for PLLA@PDA and from 52.09% to 64.45% for PLLA@PDA-DOX, indicating that NIR irradiation significantly amplifies their cytotoxic effects.

To further assess the effect on cell proliferation, EdU staining was performed, and the results are shown in Figure 5c. Blue fluorescence represents cell nuclei stained with Hoechst, while red fluorescence indicates newly synthesized DNA labeled by EdU. Compared with the PLLA and PLLA@PDA groups, the PLLA@PDA-DOX nanobubble group exhibited a markedly reduced red fluorescence signal, indicating effective inhibition of DNA synthesis and cell proliferation. In the free DOX group, the number of proliferating cells was markedly decreased, demonstrating its strong cytotoxic effect.

DOX, an anthracycline antitumor drug, exerts its cytotoxicity by intercalating into DNA, disrupting the double-helix structure, and inhibiting nucleic acid synthesis [32]. In this system, DOX is loaded onto PDA through π-π stacking or hydrogen-bond interactions and subsequently encapsulated within PLLA nanobubbles. This nanostructured delivery system reduces the direct exposure of DOX to the bloodstream, thereby lowering systemic toxicity while maintaining effective antitumor activity. Overall, the PLLA@PDA-DOX nanobubbles exhibit enhanced therapeutic efficacy through the combined effects of chemotherapy and photothermal therapy.

Compared with free DOX, PLLA@PDA-DOX nanobubbles exhibited markedly reduced cytotoxicity toward normal cells, which may be attributed to the favorable structural characteristics and biological properties of the nanosystem. First, DOX was immobilized onto the PDA layer through π-π stacking and hydrogen bonding interactions, which restrained the rapid burst release of DOX and reduced the direct acute toxicity of free drug toward normal cells. Second, the PDA-DOX complex was further encapsulated within the PLLA shell, which acted as a physical barrier to limit premature drug leakage and nonspecific exposure to normal cells. Upon ultrasonic stimulation, the PLLA@PDA-DOX nanobubbles could be disrupted to release the PDA-DOX complex, thereby enabling sustained and controlled drug release and alleviating nonspecific cytotoxicity.

In addition, owing to the suitable nanoscale size and favorable dispersion stability of PLLA@PDA-DOX nanobubbles, the nanocarriers may preferentially accumulate in tumor tissues through the EPR effect after systemic circulation, which could further reduce nonspecific drug distribution in normal tissues and improve local therapeutic efficiency [13]. Furthermore, localized photothermal heating generated by PDA under NIR irradiation may accelerate intracellular drug release and thereby enhance the combined chemo-photothermal therapeutic effect. Moreover, the PLLA shell possesses favorable biodegradability and can be gradually hydrolyzed into lactic acid in vivo, which is ultimately metabolized into carbon dioxide and water through normal physiological pathways without causing toxic accumulation [14].

### 3.3. Safety of PLLA @PDA-DOX Nanobubbles

**Figure 6:**
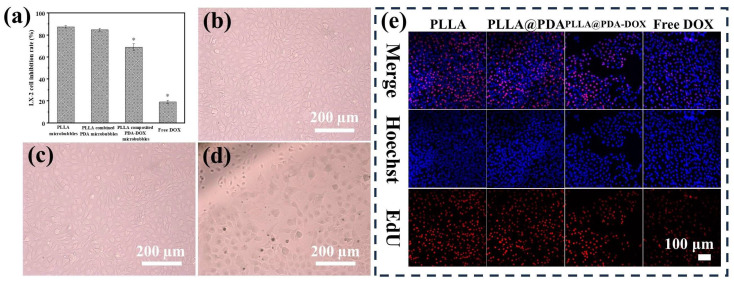
(**a**) Cell viability of LX-2 cells after 24 h of incubation with different composite nanobubbles. Data are presented as mean ± SD (n = 3) (*p* < 0.05 and * *p* < 0.01 vs. PLLA nanobubble group). (**b**–**d**) Morphological changes in LX-2 cells observed under an inverted microscope after treatment with (**b**) PLLA nanobubbles, (**c**) PLLA@PDA-DOX nanobubbles, and (**d**) free DOX. (**e**) EdU staining images of LX-2 cells after treatment with different formulations.

The cytotoxicity of different formulations toward LX-2 cells after 24 h of incubation was evaluated, and the results are shown in Figure 6a. The cell viabilities of LX-2 cells treated with PLLA nanobubbles, PLLA@PDA composite nanobubbles, PLLA@PDA-DOX nanobubbles, and free DOX were 87.4 ± 1.15%, 84.7 ± 1.19%, 68.8 ± 3.08% and 18.9 ± 1.56%, respectively. Compared with the PLLA group, the viability of LX-2 cells in the PLLA@PDA-DOX group showed a significant difference (*p* < 0.05), while that in the free DOX group exhibited a highly significant difference (* *p* < 0.01). These results indicate that free DOX induces severe cytotoxicity, whereas the PLLA@PDA-DOX nanobubbles significantly reduce the cytotoxicity of doxorubicin toward normal cells, possibly due to the controlled release behavior and encapsulation effect of the nanobubble system.

The morphological changes in LX-2 cells after different treatments are presented in Figure 6b–d. Compared with the PLLA group (Figure 6b), a slight reduction in cell number was observed in the PLLA@PDA-DOX group (Figure 6c). In contrast, in the free DOX group (Figure 6d), the number of viable cells markedly decreased, accompanied by obvious morphological damage and loss of typical cellular structure. Additional morphological observations, including the PLLA@PDA group, are provided in Figure S2. These observations further confirm that the PLLA@PDA-DOX nanobubble system reduces the cytotoxic effects of DOX.

To further evaluate cell proliferation, EdU staining was performed, and the results are shown in Figure 6e. Blue fluorescence represents cell nuclei stained with Hoechst, while red fluorescence indicates newly synthesized DNA. Strong red fluorescence signals were observed in the control group, indicating active cell proliferation. In contrast, the red fluorescence signals in the PLLA@PDA-DOX and free DOX groups were significantly reduced, suggesting effective inhibition of DNA synthesis. Notably, the inhibitory effect in the PLLA@PDA-DOX group was weaker than that of the free DOX group, indicating reduced toxicity toward normal cells.

Overall, these results demonstrate that PLLA@PDA nanobubbles exhibit good biocompatibility, and the encapsulation of DOX within the nanobubbles system significantly attenuates its cytotoxicity while maintaining its therapeutic potential.

The hemocompatibility of the nanobubbles was evaluated using a hemolysis assay, and the results are summarized in Table S3. After incubation at 37 °C for 3 h, no hemolysis was observed in the negative control group (normal saline), whereas obvious hemolysis occurred in the positive control group (distilled water). No visible hemolysis or erythrocyte agglutination was observed in the PLLA nanobubble, PLLA@PDA, or PLLA@PDA-DOX groups. The hemolysis rates of PLLA nanobubbles, PLLA@PDA composite nanobubbles, and PLLA@PDA-DOX nanobubbles were calculated to be 0.351%, 0.967% and 3.119%, respectively, all of which were below the accepted safety threshold of 5%. These results demonstrate that the prepared nanobubbles exhibit good hemocompatibility and biosafety.

The in vivo acute toxicity of PLLA@PDA-DOX nanobubbles was evaluated, and the results are summarized in Tables S4 and S5. After intraperitoneal injection at doses of 1000, 2000, and 4000 mg kg^−1^, mice in the low-dose group (1000 mg kg^−1^) exhibited normal behavior, activity, and respiration, while those in the medium-dose group (2000 mg kg^−1^) exhibited slightly reduced spontaneous activity without mortality. In contrast, mice in the high-dose group (4000 mg kg^−1^) displayed lethargy, dyspnea, and ultimately death. Based on dose–response analysis between 2000 and 4000 mg kg^−1^, the Dn and Dm were determined to be 2400 mg kg^−1^ and 3600 mg kg^−1^, respectively. The median lethal dose (LD_50_), calculated using IBM SPSS Statistics 27, was 3237.570 mg kg^−1^, with a 95% confidence interval of 3044.376~3454.732 mg kg^−1^. Specifically, one mouse died at 3000 mg kg^−1^, three mice died at 3300 mg kg^−1^, and six mice died at 3600 mg kg^−1^.

Furthermore, no mortality or obvious acute toxicity was observed at a dose of 2700 mg kg^−1^, indicating that this dose can be considered a safe threshold. The maximum tolerated dose in mice was estimated to be more than 100-fold higher than the clinical equivalent dose for humans. These results demonstrate that PLLA@PDA-DOX nanobubbles possess favorable in vivo biosafety and promising potential for clinical application.

**Figure 7:**
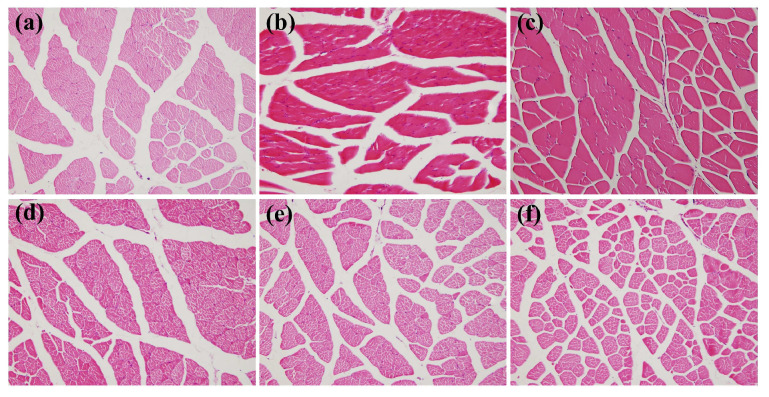
Histological images of muscle tissue sections at the injection sites of mice (200× magnification). (**a**) Control group; (**b**) 2 d; (**c**) 4 d; (**d**) 10 d; (**e**) 21 d; (**f**) 30 d.

The histological observation of muscle tissue sections at the injection sites in the normal saline control group and the experimental groups are shown in Figure 7. As seen in Figure 7a, the muscle fibers in the normal saline group were neatly arranged with no apparent signs of irritation or inflammatory response. In contrast, the muscle tissue section at 2 days after injection of PLLA@PDA-DOX nanobubbles (Figure 7b) showed a small amount of fibrin exudation from local muscle fibers. However, no significant irritation or tissue damage was observed in the samples collected at 4, 10, 21, and 30 days post-administration (Figure 7c–f). These results indicate that intramuscular injection of PLLA@PDA-DOX nanobubbles caused no obvious irritation, demonstrating their good biocompatibility.

### 3.4. In Vivo and In Vitro Ultrasonic Imaging Effects of PLLA@PDA-DOX Nanobubbles

**Figure 8:**
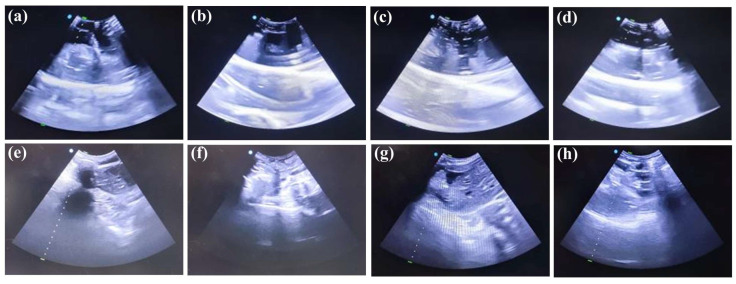
Contrast-enhanced ultrasound images of (**a**–**d**) silicone tubes in vitro and (**e**–**h**) rabbit liver in vivo after injection of saline and different nanobubbles: (**a**,**e**) saline; (**b**,**f**) PLLA nanobubbles; (**c**,**g**) PLLA@PDA nanobubbles; and (**d**,**h**) PLLA@PDA-DOX nanobubbles.

Figure 8 presents the in vitro and in vivo ultrasound imaging performance of different formulations. The in vitro ultrasound contrast images are shown in Figure 8a–d. Compared with the normal saline group (Figure 8a), the PLLA nanobubble, PLLA@PDA nanobubble, and PLLA@PDA-DOX nanobubble groups (Figure 8b–d) exhibited significantly enhanced ultrasound imaging signals. No significant differences in echo intensity were observed among the three nanobubble groups, indicating that the introduction of PDA and DOX did not affect the ultrasound imaging performance or signal stability of PLLA nanobubbles.

The in vivo ultrasound imaging results of rabbit livers are presented in Figure 8e–h. In the normal saline group (Figure 8e), the echo signal was weak and the liver boundary was indistinct. In contrast, the PLLA nanobubble group (Figure 8f) showed markedly enhanced echo intensity and improved image brightness. In the PLLA@PDA and PLLA@PDA-DOX groups (Figure 8g,h), the size, shape, and internal echo structure of the liver were clearly visualized, demonstrating improved imaging clarity and resolution. Furthermore, the echo intensities of the PLLA@PDA and PLLA@PDA-DOX groups were comparable, indicating that DOX loading did not compromise the ultrasound contrast performance of the nanobubbles.

Overall, these results demonstrate that PLLA@PDA-DOX nanobubbles possess excellent ultrasound imaging performance both in vitro and in vivo, while maintaining stable contrast performance after functional modification.

The in vivo ultrasound imaging performance of PLLA@PDA-DOX nanobubbles at 1 h post-injection is shown in Figure S3. No significant attenuation or quenching of the ultrasound signal was observed after 1 h, and the position and outline of the rabbit liver remained clearly visible. These results indicate that the PLLA@PDA-DOX nanobubbles possess good in vivo stability and are capable of maintaining prolonged and consistent imaging performance.

## 4. Conclusions

Based on the excellent photothermal conversion capability and biocompatibility of PDA, the chemotherapeutic agent DOX was successfully loaded onto the surface of PDA through π–π stacking and hydrogen bonding interactions. The prepared PLLA@PDA-DOX nanobubbles exhibited a uniform particle size of 489.30 ± 6.96 nm, favorable ultrasound imaging performance, and remarkable photothermal performance under NIR irradiation. Notably, the temperature of the PLLA@PDA-DOX nanobubble suspension increased from 22.8 °C to 44.6 °C after 10 min of laser irradiation, exceeding the effective threshold for tumor cell ablation. In addition, the photothermal performance remained nearly unchanged after three irradiation cycles, demonstrating excellent photothermal stability. Under NIR irradiation, the inhibition rate of HCCLM3 cells increased from 50.1% to 64.45%, indicating enhanced antitumor efficacy through combined chemo-photothermal therapy.

Although DOX was successfully incorporated into the PLLA@PDA-DOX nanobubble system, direct drug release kinetics, particularly under NIR irradiation, were not evaluated in the present study. Future studies will therefore focus on investigating the NIR-responsive drug release behavior, as well as the pharmacokinetic characteristics, and long-term toxicity of the developed nanobubble system to further assess its clinical translational potential. Therefore, the multifunctional PLLA@PDA-DOX nanobubble system developed in this study shows promising potential for ultrasound imaging-guided cancer theranostics.

## Data Availability

The original contributions presented in this study are included in the article/Supplementary Materials. Further inquiries can be directed to the corresponding author.

## Supplementary Materials

The following supporting information can be downloaded at: https://www.mdpi.com/article/10.3390/biom16060834/s1, Table S1. Contents of hemolysis test in each test tube. Table S2. Drug loading rate of DOX in PLLA@PDA-DOX nanobubbles (n = 3). Table S3. Experimental results of hemolysis induced by different nanobubbles. Table S4. Acute toxicity test results of PLLA@PDA-DOX nanobubbles in mice. Table S5. Safety limit test results of PLLA@PDA-DOX nanobubbles in mice. Figure S1. Standard calibration curve of DOX. Figure S2. Morphological changes of LX-2 cells observed under an inverted microscope after treatment with PLLA@PDA nanobubbles. Figure S3. Ultrasound images of rabbit liver 1 h after injection of PLLA@PDA-DOX nanobubbles.
