# Supplementary material for: PLLA@PDA-DOX Nanobubbles for Ultrasound Imaging Combined Chemo-Photothermal Therapy
Source: Biomolecules. 2026 Jun 4;16(6):834. doi: 10.3390/biom16060834 (PMC13296396; doi:10.3390/biom16060834)
Supplement: Supplementary file 1 [file biomolecules-16-00834-s001.zip › biomolecules-4304661-supplementary.pdf]

Supplementary Materials

Table S1. Contents of hemolysis test in each test tube.

Test tube number	1(ODE)	2(ODE)	3(ODE)	4(ODNC)	5(ODPC)
Red blood cell suspension(mL)	0.2	0.2	0.2	0.2	0.2
Normal saline(mL)	10	10	10	10	0
Deionized water(mL)	0	0	0	0	20
Different groups of nanobubbles	PLLA nanobubbles	PLLA@PDA nanobubbles	PLLA@PDA-DOX nanobubbles	no	no

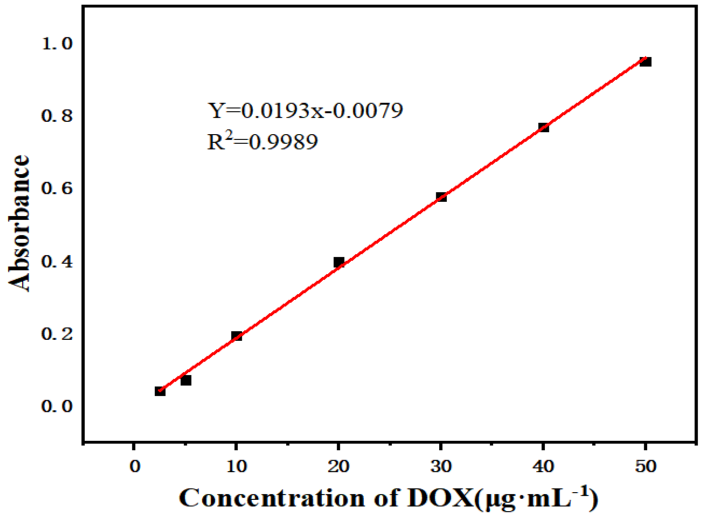

Figure S1. Standard calibration curve of DOX.

Table S2. Drug loading rate of DOX in PLLA@PDA-DOX nanobubbles (n = 3).

Number	Absorbance	Loading rate (%)
1	0.623	3.27
2	0.619	3.25
3	0.627	3.29
Average	0.623	3.27

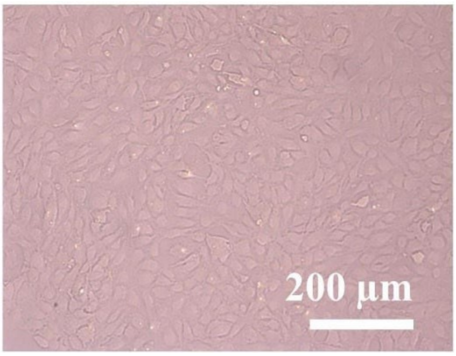

Figure S2. Morphological changes of LX-2 cells observed under an inverted microscope after treatment with PLLA@PDA nanobubbles.

Table S3. Experimental results of hemolysis induced by different nanobubbles.

OD Value	PLLA nanobubbles	PLLA@PDA nanobubbles	PLLA@PDA-DOX nanobubbles	Negative control group	Positive control group
1	0.115	0.108	0.123	0.106	0.566
2	0.115	0.11	0.125	0.105	0.561
3	0.108	0.113	0.12	0.107	0.555
4	0.102	0.117	0.123	0.12	0.565
5	0.109	0.115	0.121	0.103	0.57
Mean value	0.1098	0.1126	0.1224	0.1082	0.5635
Hemolysis rate (%)	0.351	0.967	3.119	0	100
Result	-	-	-	-	+

Note:"+" indicates hemolysis or aggregation,"-"indicates no hemolysis or aggregation.

Table S4. Acute toxicity test results of PLLA@PDA-DOX nanobubbles in mice.

Group	Dose （mg kg^{-1}）	Logarithmic dose	Number of animals	Number of death	Death rate
1	2400	3.380	6	0	0
2	2700	3.341	6	0	0
3	3000	3.477	6	1	0.16
4	3300	3.519	6	3	0.5
5	3600	3.556	6	6	1

Table S5. Safety limit test results of PLLA@PDA-DOX nanobubbles in mice.

Group	Species	Sex	Abnormal behavior	Abnormal body weight	Observation time (d)	Death
1	KM	male	no	no	14	no
2	KM	male	no	no	14	no
3	KM	male	no	no	14	no
4	KM	male	no	no	14	no
5	KM	male	no	no	14	no
6	KM	female	no	no	14	no
7	KM	female	no	no	14	no
8	KM	female	no	no	14	no
9	KM	female	no	no	14	no
10	KM	female	no	no	14	no

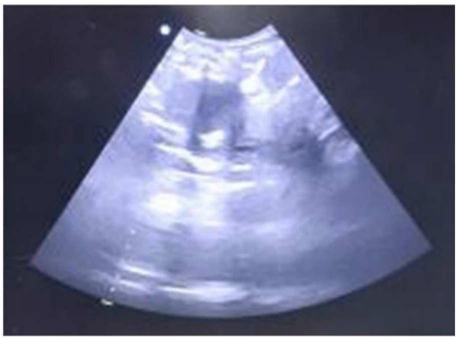

Figure S3. Ultrasound images of rabbit liver 1 h after injection of PLLA@PDA-DOX nanobubbles.
